# In vivo effect of interleukin-1beta and interleukin-1RA on oocyte cytoplasmic maturation, ovulation, and early embryonic development in the mare

**DOI:** 10.1186/1477-7827-3-26

**Published:** 2005-06-22

**Authors:** Maud Caillaud, Guy Duchamp, Nadine Gérard

**Affiliations:** 1 Physiologie de la Reproduction et des Comportements. INRA-CNRS-Université de Tours-Haras Nationaux, IFR135, 37380 Nouzilly France

## Abstract

A growing body of evidence suggests that the interleukin-1 system is involved in periovulatory events. Previous work from our lab demonstrated that in the mare, interleukin-1beta (IL-1beta) increases the ovulatory rate of metaphase II oocytes. The present study was conducted to analyze in vivo the effect of IL-1 on oocyte cytoplasmic maturation, ovulation and pregnancy rate. In the present work, IL-1beta (experiment 1, n = 13; experiment 2, n = 25) and interleukin-1RA (IL-1RA; experiment 1, n = 25) were injected intrafollicularly by using the transvaginal ultrasound-guided injection method. Injections were performed on cyclic mares when the diameter of the growing dominant follicle reached 30–34 mm. In experiment 1, mares were inseminated the day of the treatment and all the other day until ovulation. The time of ovulation was determined and a pregnancy diagnosis was performed 14 days after ovulation of the injected follicle. In experiment 2, the cumulus-oocyte complex from each injected follicle was collected by transvaginal ultrasound-guided aspiration 38 h after the intrafollicular injection. Oocyte nuclear stage and oocyte cytoplasmic maturation were assessed by analyzing chromatin configuration, cortical granules migration and mitochondria distribution under a confocal microscope. The results from experiment 1 confirm that an intrafollicular injection of 1 microgram IL-1beta induces ovulation in the mare whereas IL-1RA has no effect at the dose used in the present study. Furthemore, we demonstrated, that in our experimental conditions, IL-1beta and IL-1RA induced a decrease in embryo development. Experiment 2 leads us to observe that IL-1beta is unable to induce cortical granules migration and remodelling of mitochondria, that commonly occurs during oocyte maturation, whereas it acts on nuclear maturation. This result may explain the decrease in embryo development we observed after IL-1beta intrafollicular injection. In conclusion, the present study tends to demonstrate that IL-1beta plays a role in the ovulatory process and may acts on oocyte maturation in the mare, but additional factors are required to complete equine oocyte cytoplasmic maturation to allow embryo development.

## Background

In recent years, evidence has accumulated to suggest that cytokines are important regulators of the ovarian function. Interleukin-1 (IL-1), one major cytokine, as well as receptors (IL-1R1 and IL-1R2) and antagonist (IL-1RA) have been demonstrated to be produced in the ovary of several species [1, for review]. Recently, we demonstrated the presence of IL-1β mRNA in equine cumulus-oocyte complexes and granulosa cells, whereas immunoreactive IL-1β has been observed in equine follicular fluids [2,3].

In vitro studies have shown that IL-1β regulates some cellular activities of granulosa and theca cells, such as steroidogenesis [4,5], as well as synthesis of proteases [6-8] and prostaglandins [9,10]. Moreover, it has been demonstrated that IL-1β promotes the ovulation process in the rat [11], the rabbit [12] and the mare [13]. IL-1β increases in vitro the germinal vesicle breakdown of oocytes in the rabbit model [12], as well as in vivo in the mare [13], demonstrating a beneficial role of IL-1β in oocyte nuclear maturation. These observations led to conclude that IL-1 may be a paracrine factor that is involved in ovulation and in oocyte nuclear maturation. In 1994, Takehara *et al*., demonstrated that IL-1β can facilitate in vitro fertilization in the rabbit, suggesting that IL-1 may be involved in oocyte cytoplasmic maturation and could improve fertilization. In the mare, no data is available on the effect of IL-1β on oocyte cytoplasmic maturation or fertilization rates. Nevertheless, a better knowledge of regulating factors involved in these processes would help to improve the embryo production in this species.

In this context, the objectives of the present study were to investigate in vivo in the mare, the potential role of IL-1β on oocyte cytoplasmic maturation, ovulation and embryo development.

## Materials and methods

### Animals and Treatments

All animal procedures were approved by the Agricultural Agency and Scientific Research Agency (approval number A37801) and conducted in accordance with guidelines for Care and Use of Agricultural Animals in Agricultural Research and Teaching.

Adult cyclic pony mares in good body condition, kept indoors and fed with concentrates, were used. Mares received a prostaglandin F2α analogue (Cloprostenol [estrumate], 125 μg/mare i.m.; Scherring-Plough, Levallois-Peret, France) during the midluteal phase to induce luteolysis. Ovarian activity was then assessed by routine rectal ultrasound scanning. Intrafollicular injections were performed in the dominant follicle, between 30–34 mm in diameter, at the end of the follicular phase (see below). Before intrafollicular injections or punctures, the mares were sedated with detomidine (0.6 mg/100 kg body weight [BW] i.v., Domosedan; Pfizer, Amboise, France) and propantheline bromide (3 mg/100 kg BW i.v.; Sigma, St. Louis, MO) was administered to achieve rectal relaxation. After intrafollicular injection or punctures, mares were treated with an antibiotic (Intramicine, 1 600 000 IU penicillin/100 kg BW and 1.3 g dihydrostreptomycin/100 kg BW i.m.; Sanofi, Libourne, France).

Crude Equine Gonadotropin (CEG) was prepared from horse pituitaries that were supplied commercially. The resulting preparation of CEG contained approximately 6% horse LH and 2–4% horse FSH. An i.v. injection of 15 mg CEG was performed when the diameter of the dominant follicle reached 30–34 mm in order to induce ovulation. Ovulation occurs between 35–40 hours after the injection [14]

### Intrafollicular Injection Procedure

The injection was performed using a transvaginal ultrasound-guided system. A 60-cm-long single lumen needle of 1.1-mm outer diameter was inserted into the dominant follicle the day it reached 30–34 mm in diameter. Follicular fluid (2.5 ml) was aspirated in a syringe directly connected to the needle. The studied molecule was diluted in 2 ml of PBS (Dulbecco 'A'; Unipath, Dardilly, France) and maintained at 37°C. The 2 ml were then injected in the follicle by using a syringe directly connected to the needle. Follicular fluid (0.5 ml) from the first syringe was then injected back into the follicle to rince the needle and reestablish the initial follicular volume.

### Experimental Designs

In experiment 1, 61 cyclic mares were used. They were divided into five groups. Mares from the first group (negative control group; n = 12) received an intrafollicular injection (i.f.) of 2 ml of PBS and an jugular injection (i.v.) of 5 ml of saline. Mares from the second group (IL-1RA group; n = 12) received an i.f. injection of 2 ml of recombinant human interleukin-1RA (2.5 μg/ml in PBS, rhIL-1RA; R&D System, Abingdon, UK) and an i.v. injection of 5 ml of saline. Mares from the third group (IL-1β group; n = 13) received an i.f. injection of 2 ml of recombinant human IL-1β (0.5 μg/ml in PBS, rhIL-1β; R&D System) and an i.v. injection of 5 ml of saline. Mares from the fourth group (positive control group; n = 12) received an i.f. injection of 2 ml of PBS and an i.v. injection of 15 mg CEG in 5 ml of saline. Mares from the last group (IL-1RA/CEG group; n = 13) received an i.f. injection of 2 ml of recombinant human interleukin-1RA (2.5 μg/ml in PBS, rhIL-1RA) and an i.v. injection of 15 mg CEG in 5 ml of saline.

In experiment 2, 63 cyclic mares were used. They were divided into three groups. Mares from the first group (positive control group; n = 23) received an i.f. injection of 2 ml of PBS and an i.v. injection of 15 mg CEG in 5 ml of saline. Mares from the second group (IL-1β group; n = 25) received an i.f. injection of 2 ml of recombinant human IL-1β (0.5 μg/ml in PBS, rhIL-1β; R&D System) and an i.v. injection of 5 ml of saline. Mares from the last group (negative control group; n = 15) received an i.f. injection of 2 ml of PBS and an i.v. injection of 5 ml of saline.

### Artificial insemination, Detection of Ovulation and Pregnancy Diagnosis

Mares from experiment 1 were inseminated with fresh semen collected from a fertile stallion, using 200 × 10^6 ^spermatozoa per insemination. Inseminations were performed on the day of the intrafollicular injection, on the day after and every 2 days until ovulation of the injected follicle. Ovulation was assessed ultrasonographically twice a day from 8 h after the intrafollicular injection until ovulation (i.e., absence of a large dominant follicle and presence of a corpus luteum). Fourteen days after ovulation, pregnancy diagnosis was performed by routine ultrasonography of the uterus.

### Cumulus Oocyte Complex (COC) Recovery and Analysis

COCs were collected from injected follicles of experiment 2. Transvaginal ultrasound-guided aspiration was used 38 h after the intrafollicular injection, as previously described [15]. Briefly, a single lumen needle (1.8-mm outer diameter) was used to aspirate follicular fluid. The follicle was then flushed several times with PBS containing heparin (2.5 IU/ml; LEO S.A., St Quentin Yvelines, France) at 37°C and scraped with the needle in order to pick up the oocyte. All aspirated fluids were examined with a stereomicroscope for oocyte recovery. Cumulus cells were removed from the oocytes by repeated pipetting and subsequent treatment with 1% hyaluronidase (type III, 875IU/mg; Sigma, La Verpillère, France). Oocytes were then incubated for 30 min at 37°C in PBS containing 3% BSA and 280 nM MitoTracker Orange CMTM Ros (Molecular Probes, Oregon, USA) for mitochondria detection. Then, oocytes were washed three times in pre-warmed PBS without BSA. The oocytes were then fixed for 20 min at 37°C using freshly prepared 2.5 % paraformaldehyde in PBS. After fixation, the oocytes were washed three times in PBS. The oocytes were then permeabilized in Triton X-100 (Sigma), 0.1% in PBS, for 5 min at room temperature and washed in PBS. Oocytes were then incubated for 30 min at room temperature in 100 μg/ml of fluorescein isothiocyanate-conjugated peanut agglutinin in washing solution (PBS containing 0.05 % NaN_3 _and 1 mM PMSF) to detect distribution of cortical granules. After rinsing, oocytes were stained with 1 μg/ml *bis*-benzimide (Hoechst 33342; Sigma) in PBS for 6 min at room temperature for DNA detection. They were then mounted between slide and cover slide in a mixture of Moviol V4–88 (133 mg/ml; Hoechst, Frankfurt, Germany) and *n*-propyl gallate (5 mg/ml; Sigma). The slides were kept at 4°C in darkness until observation. The oocytes were observed under a confocal laser scanning microscope (Olympus, France). Mitochondria and cortical granules labelling were visualized in the confocal mode, whereas Hoechst staining was detected by conventional epifluorescence. For fluorescein, an argon ion laser adjusted at 525-nm emission wave length was used; for MitoTracker Orange CMTM Ros, a helium-neon ion laser adjusted at 576-nm was used.

### Statistical analysis

The nonparametric tests of Kruskal-Wallis and Wilcoxon-Mann-Withney were performed using StatXact 5 software (CYTEL, Cambridge, MA ) in order to compare time of ovulation and percentage of embryos. The corrected chi-square test was used to compare oocyte in vivo maturation rates.

## Results

### Experiment 1: Effect of IL-1β and IL-1RA intrafollicular injection on ovulation and embryo development

During this experiment, 61 follicles were injected. Twelve to thirteen mares were used per group, in 5 groups.

#### Time of ovulation

**Figure 1:**
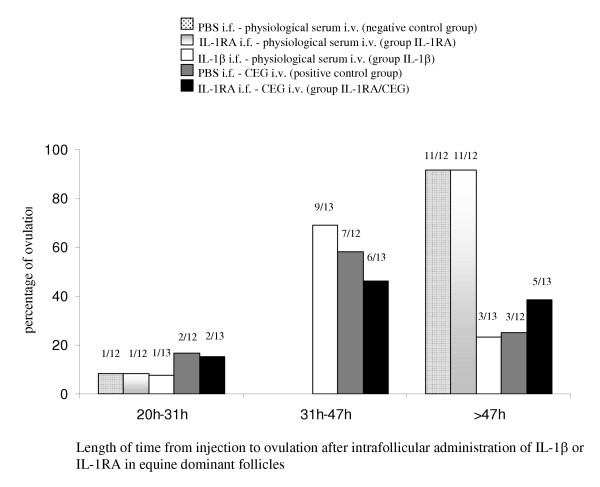
Length of time from injection to ovulation after intrafollicular administration of IL-1β or IL-1RA in equine dominant follicles. For each group, histograms represent the percentage of mares that ovulated during each time interval. The proportion of mares is indicated on the top of each histogram. The distribution of the time of ovulation was not significantly different between the positive control group and the IL-1β group. In the negative control group and in the IL-1RA group, the distribution of the time of ovulation was significantly different from that in the positive control group and in the IL-1β group. In the IL-1RA/CEG group, this distribution was not significantly different from that in the positive control group and the IL-1β group but differed significantly from that in the negative control group and in the IL-1RA group.

Time of ovulation after intrafollicular injection of PBS, IL-1β or IL-1RA was determined by ultrasonography. We determined three intervals during which the mares ovulated: between 20 and 31 h, between 31 and 47 h, and more than 47 h after the intrafollicular injection. Figure 1 illustrates the ovulation profiles after intrafollicular injection of PBS (2 ml), IL-1β (1 μg/2 ml), or IL-1RA (5 μg/2 ml). In the positive control group (PBS i.f., CEG i.v.), mares ovulated mainly between 31 and 47 h after the intrafollicular injection (7/12). Mares from the IL-1β group (IL-1β i.f., saline i.v.) ovulated mainly in the same interval (9/13). The distribution of the time of ovulation was not significantly different between these two groups. In the negative control group (PBS i.f., saline i.v.), most of the mares ovulated more than 47 h after the i.f. injection (11/12). The IL-1RA group (IL-1RA i.f., saline i.v.) displayed a similar distribution of time of ovulation (11/12 ovulations after 47 h). This distribution was significantly different from that of the positive control group (p < 0.002) and IL-1β group (p < 0.001). In the IL-1RA/CEG group (IL-1RA i.f., CEG i.v.), the length of time from injection to ovulation was heterogeneous. Half of the mares (6/13) ovulated between 31 and 47 h after the i.f. injection, whereas 2/13 ovulated during the first interval (20–31 h) and 5/13 ovulated more than 47 h after the i.f. injection. This distribution was not significantly different from that of the positive control group and IL-1β group but differed significantly from the negative control group and the IL-1RA group (p < 0.01 each).

#### Embryo development

Presence or absence of an embryo was determined in each mare by ultrasonography 14 days after ovulation. Table 1 illustrates the percentage of embryos observed after intrafollicular injection of PBS (2 ml), IL-1β (1 μg/2 ml), or IL-1RA (5 μg/2 ml) coupled to an intraveneous injection of either saline or CEG. The percentage of embryos was the same in the two control groups (67%). In contrast, the percentage of embryos was lower in the three other groups (25%, 23%, 23%) which received IL-1β or IL-1RA intrafollicularly. These lower percentages are not statistically different from one to the other but are significantly different from that of both control groups (p < 0.05).

**Table 1:** Percentage of embryos after intrafollicular administration of IL-1β or IL-1RA in equine dominant follicles.

	Number of injected follicles	Percent of embryos
PBS i.f., saline i.v. (negative control group)	12	67 (8/12)^a^
IL-1RA i.f., saline i.v. (IL-1RA group)	12	25 (3/12)^b^
IL-1β i.f., saline i.v. (IL-1β group)	13	23 (3/13)^b^
PBS i.f., CEG i.v. (positive control group)	12	67 (8/12)^a^
IL-1RA i.f., CEG i.v. (IL-1RA/CEG group)	13	23 (3/13)^b^

PBS, Phosphate buffer saline; CEG, crude equine gonadotropin; IL-1β, interleukin 1 beta; IL-1RA, interleukin 1 receptor antagonist; i.f., intrafollicular; i.v., intravenous. a and b are significantly different (p < 0.05).

### Experiment 2: Effect of IL-1β on oocyte nuclear and cytoplasmic maturation

Cumulus Oocyte Complex recovery and Oocyte nuclear stage at recovery

In this experiment, 3 groups were constituted: positive control group, IL-1β group, negative control group. Twenty three to 25 mares were used in the 2 first groups and 15 mares were used in the third group. Out of the sixty three injected follicles that were punctured, 52 oocytes were collected and analyzed. This corresponds to 82.5% of collection rate. The collection rate was similar in each group (table 2). All aspirated COCs had expanded cumuli.

As shown in Table 2, the rate of oocytes that display resumption of meiosis was significantly higher in the positive control group (PBS i.f., CEG i.v.) than in the negative control group (PBS i.f., saline i.v.; p < 0.04). The other oocytes were either immature or degenerated. Moreover, 60% of oocytes from the IL-1β group display resumption of meiosis, that tends (p < 0,1) to be higher than that in the negative control group.

**Table 2:** Number of punctured follicles, percentage of recovered cumulus-oocyte complexes, and percentage of degenerated, immature, metaphase I and metaphase II oocytes in each group.

		COCs	Oocytes		
		
	Number of injected-punctured follicles	Percent of recovery	Percent of DEG	Percent of immature	Resumption of meiosis
PBS i.f., CEG i.v.	23	91 (21/23)^a^	19 (4/21)	0 (0/21)	81 (17/21)^a^
IL-1β i.f., saline i.v.	25	80 (20/25)^a^	20 (4/20)	20 (4/20)	60 (12/20)^ab^
PBS i.f., saline i.v.	15	73 (11/15)^a^	27 (3/11)	27 (3/11)	45 (5/11)^b^

PBS, Phosphate buffer saline; CEG, crude equine gonadotropin; IL-1β, interleukin 1 beta; i.f., intrafollicular; i.v., intravenous; DEG, degenerated; MI metaphase I; MII, metaphase II. a and b are significantly different (P < 0.05).

#### Cortical granules localization and Mitochondria distribution

**Figure 2:**
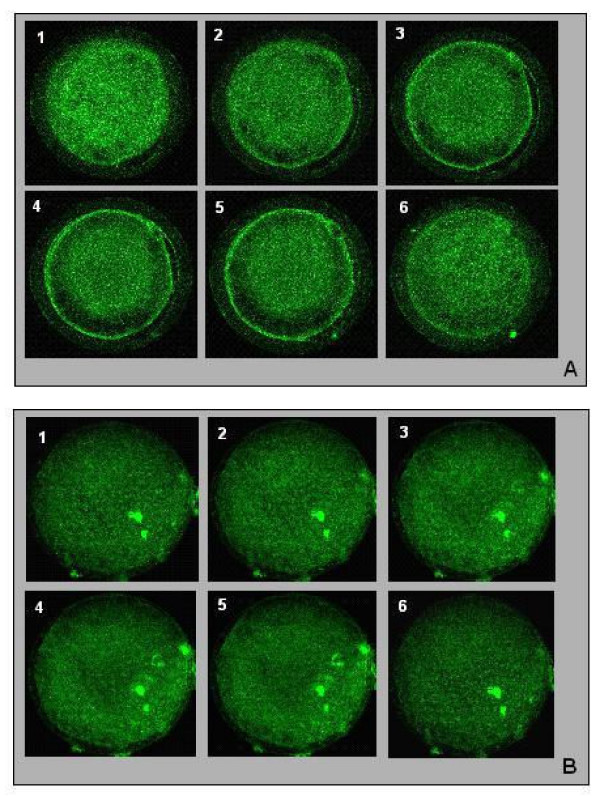
Cortical granules localization on serial optical sections in equine oocytes after intrafollicular injection of PBS (A) or IL-1β (B) and intravenous injection of CEG (A) or saline (B). 1–6: different sections from the top of the oocyte to the bottom of the oocyte **A**) Cortical granules migration achieved: the majority of the cortical granules are lining the oolemma. **B**) No cortical granules migration: the cortical granules are located in the medullary zone, and no cortical granules are lining the oolemma; X40

Mature (MII) oocytes were analyzed in order to visualize cortical granules and mitochondria. Thirteen matures oocytes from the positive control group were analyzed. Cortical granules migration was achieved or in progress in most oocytes from this group (8/13), with cortical granules lining the oolemma (Figure 2A). In the 5 other oocytes from this group, cortical granules were located in the medullary zone. In 3 to 5 mature oocytes analyzed from the IL-1β group, cortical granules had an uniform distribution between the oolemma and the medullary zone (Figure 2B), and only 2 oocytes displayed cortical granules migration in progress.

In most oocytes from the positive control group (9/13), the distribution of mitochondria was heterogeneous i.e. labelled mitochondria were distributed unevenly within the cytoplasm (data not shown). Mitochondria distribution in oocytes from the IL-1β group was more homogeneous with labelled mitochondria distributed evenly throughout the cytoplasm (data not shown).

## Discussion

The aim of the present work was to analyze in vivo the effect of IL-1β and IL-1RA, after their intrafollicular injection into the preovulatory follicle. In experiment 1, ovulation and embryo development were studied. In experiment 2, the effect of IL-1β on oocyte nuclear and cytoplasmic maturation was investigated.

The intrafollicular injection technique is an interesting alternative to study the effect of a factor on oocyte and follicle maturation. It has been used in different species like rabbit [16], ewe [17], cow [18], rhesus monkey [19], and mares [13,15,20,21]. In the mare, this approach needs no surgery because the large size of the preovulatory follicle and the presence of a tunica albuginea, which allows injecting follicles with limited leakage by using the transvaginal ultrasound echo-guided method. In the present work, 103 preovulatory follicles were successfully injected and only 5 suffered serious damage.

In our study, 1 μg IL-1β was injected in the follicle. This dose was chosen regarding a dose-dependent study that had been performed in vitro [22], and tested in vivo [13]. Similarly, the dose of IL-1RA we used in the present study (5 μg) was chosen in accordance to a previous in vivo study [13] where 1 μg IL-1RA had no effect.

We demonstrated in the present work, that the intrafollicular injection of IL-1β induced synchronized ovulations. Actually, IL-1β gave the same result, in terms of ovulation distribution, as an i.v. injection of CEG [14]. This observation is in accordance with the recent work performed by Martoriati *et al*. [13]. CEG is a pituitary extract used to induce ovulation in the mare. By its LH activity, CEG is used to give a precise timing of ovulation. It is preferentially used in this specie instead of hCG, which is known to induce immunoreaction after successive injections. Although, the mechanisms by which IL-1 regulates follicular maturation is unclear, our observations suggest that IL-1 system could be involved in periovulatory events. Thus, the mechanism of action of IL-1β remains to be elucidated, but we can hypothesize that the increase in IL-1β intrafollicular level after injection may mimics the local preovulatory events that precede ovulation.

That hypothesis explains why we constituted the IL-1RA-CEG group, in which the intrafollicular injection of IL-1RA would be able to block the CEG effect. After such treatment (IL-1RA i.f. and CEG i.v.), we observed that half of mares ovulated more than 47 h after injections. In such mares, we can conclude either that CEG had no effect (that is unlikely at this rate), or that the intrafollicular injection of IL-1RA at the dose we used, inhibited the ovulatory effect of CEG. Other mares from this group ovulated between 31 and 47 h after injection. In these mares and in our experimental conditions, IL-1RA did not inhibit the CEG ovulatory effect. These mares would probably had the process of ovulation engaged (i.e. increase in the endogenous plasmatic concentration of LH) before IL-1RA and CEG injections. The data we obtained from the IL-1RA-CEG group are in accordance with a previous study performed in the rat, that showed a time-dependent inhibition of hCG-induced ovulation by IL-1RA [23].

As demonstrated in the present work, 60% of the oocytes collected 38 h after IL-1β intrafollicular injection displayed resumption of meiosis. This rate only tends to be higher than that of the negative control group (p < 0,1). This result is consistent with our previous result obtained in the mare (57%) [13]. Taken together, both studies imply a positive effect of IL-1β on equine oocyte nuclear maturation.

Considering this result, it was then questionable if ovulated mature equine oocytes obtained after an intrafollicular injection of IL-1β were able to be fertilized. The very low rate of embryo development that we observed in our study after IL-1β intrafollicular injection (25%) leads us to hypothesize that IL-1β may have a negative effect on oocyte quality.

We tested this hypothesis by analyzing metaphase II (MII) oocytes for cortical granules localization and mitochondria distribution as criteria for cytoplasmic maturation. To our knowledge, this is the first study of the effect of IL-1β on oocyte cytoplasmic maturation that has been performed in vivo in any species. In several mammals, it has been reported that cortical granules are mainly lining the oolemma of oocytes at the time of ovulation (in sheep [24] ; in bovine [25-27] ; in equine [28]). In our study, cortical granules migration was achieved or in progress in most MII oocytes from the positive control group (CEG group). In contrast, cortical granules did not line the oolemma in most MII oocytes from IL-1β injected follicle, but remained homogeneously distributed in the cytoplasm. This data suggests that in our study, IL-1β was unable to induce cortical granules migration, and thus, to be fully involved in oocyte cytoplasmic maturation. Additional factors may be required for cortical granules migration.

The distribution of mitochondria in MII oocytes was studied as a second criterion of cytoplasmic maturation. Two mitochondrial distribution patterns were observed in equine oocytes. The oocytes from the positive control group (CEG group) displayed a clustering of mitochondria around cytoplasmic vacuoles (heterogeneous pattern), which observation is in accordance with previous studies (bovine [29] ; human [30,33,34] ; porcine [31,32]). The second mitochondrial distribution pattern that we observed mainly concerned oocytes from the IL-1β group. In these oocytes, mitochondria displayed a homogeneous distribution. Such a homogeneous pattern of mitochondria had previously been observed in immature porcine oocytes [32]. This data suggests that in our experimental conditions, IL-1β is unable to induce the remodelling of mitochondria that occurs during oocyte maturation.

All in all, whereas IL-1β seems to be able to act on resumption of meiosis, it is not sufficient for complete oocyte cytoplasmic maturation, at least in the mare.

To be fertilized, an oocyte has to be mature up to his nucleus and his cytoplasm. Thus, we could propose that the low number of embryos conceived after IL-1β intrafollicular injection could be probably due to an abnormal cytoplasmic maturation of oocytes. This result seems at odds with a previous study performed on perfused rabbit ovary, in which IL-1β facilitated fertilization [12]. However, these authors reported that most of fertilized oocytes were arrested at the four-cell stage. Regarding this findings, two hypothesis can be proposed to explain our results. The first one is that IL-1β is able to act on resumption of meiosis, but cannot bring about cytoplasmic maturation. The consequence is that oocytes are not suitable for fertilization. The second hypothesis is that IL-1β may exert a cytotoxic effect on oocytes, that would allow fertilization but prevent subsequent embryonic development. In our study, pregnancy diagnosis was performed 14 days after ovulation, that do not allow to verify one of these two hypothesis. To alleviate this problem, in vitro fertilization (IVF) could be performed on IL-1β-induced matured oocyte.

## Conclusion

In conclusion, the mechanism by which IL-1β acts at the ovarian level is unclear, and the present data tend to demonstrate that IL-1β alone is not able to promote cytoplasmic maturation of equine oocyte, and thus to allow embryo development. Nevertheless, IL-1β may play an essential role in the physiology of equine oocytes by acting on meiosis resumption as well as in the ovarian function by inducing ovulation.

## Authors' contributions

MC participated to the conception of the study, coordinated and participated to the experimental procedures, analyzed results and drafted the manuscript. GD carried out and coordinated the experimental procedures. NG conceived the study, participated in its design and to the experimental procedures. NG participated to the final analysis of results and corrected the manuscript. All the authors read and approved the final manuscript.

## Financial support

This work was supported by grants from the Institut National de la Recherche Agronomique, France, and the Haras Nationaux, France. Maud Caillaud was supported by a fellowship from the Région Centre, France and the Haras Nationaux, France.
